# An equivalent condition to the Jensen inequality for the generalized Sugeno integral

**DOI:** 10.1186/s13660-017-1561-2

**Published:** 2017-11-14

**Authors:** Mohsen Jaddi, Ali Ebadian, Manuel de la Sen, Sadegh Abbaszadeh

**Affiliations:** 1 Department of Mathematics, Payame Noor University, Tehran, 19395-4697 Iran; 2 Department of Electricity and Electronics, University of the Basque Country, Bilbao, 466, 48080 Spain

**Keywords:** 26D15, 28A25, generalized Sugeno integral, convex function, the Jensen inequality

## Abstract

For the classical Jensen inequality of convex functions, i.e.,

$$\begin{aligned} H \biggl(\frac{1}{\mu(D)} \int_{D}f\,d\mu \biggr)\leq\frac{1}{\mu (D)} \int_{D} H\circ f\,d\mu, \end{aligned}$$

an equivalent condition is proved in the framework of the generalized Sugeno integral. Also, the necessary and sufficient conditions for the validity of the discrete form of the Jensen inequality for the generalized Sugeno integral are given.

## Introduction

The classical Jensen inequality is one of the interesting inequalities in the theory of differential and difference equations, as well as other areas of mathematics. The well-known Jensen inequality for a convex function is given as follows:

Let $(X,\Sigma,\mu)$ be a measure space, *f* be a real-valued *μ*-measurable and *μ*-integrable function on a set $D\in\Sigma$ with $\mu(D)\in{]}0,\infty[ $. If *φ* is a convex function on an open interval *I* in $\mathbb{R}$ and if $f(D)\subset I$, then

1 $$ H \biggl(\frac{1}{\mu(D)} \int_{D}f\,d\mu \biggr)\leq\frac{1}{\mu (D)} \int_{D} H\circ f \,d\mu. $$

In recent years, there have been many extensions, refinements and similar results of the classical Jensen inequality, see [1–5].

The concepts of fuzzy measures and the Sugeno integral were introduced and initially examined by Sugeno [6]. Further theoretical investigations of these concepts and their generalizations have been pursued by many researchers. Among them, Ralescu and Adams [7] provided several equivalent definitions of the Sugeno integral and proved a monotone convergence theorem for the Sugeno integral; Román-Flores et al. [8, 9] discussed level-continuity of the Sugeno integral and *H*-continuity of fuzzy measures, while Wang and Klir [10] presented an excellent general overview on fuzzy measurement and fuzzy integration theory. The Sugeno integral has also been successfully applied to various fields (see, e.g., [11–14]).

The study of inequalities for the Sugeno integral was initiated by Román-Flores et al. [15]. Since then, fuzzy integral counterparts of several classical inequalities, including the Chebyshev, Jensen, Minkowski, Hadamard and Hölder inequalities, have been presented (see [1–3, 16–18]).

Kaluszka et al. [2] studied the Jensen inequality (1) for the generalized Sugeno integral by using the condition of monotonicity instead of the condition of convexity. The aim of this paper is to study the Jensen inequality for the generalized Sugeno integral without losing the condition of convexity.

The paper is organized as follows. Some basic definitions and summarizations of previous results are given in Section 2. In Section 3, the Jensen inequality for the generalized Sugeno integral is studied. In Section 4, the necessary and sufficient conditions for the validity of the discrete form of the Jensen inequality is presented. A conclusion is given in Section 5.

## Preliminaries

In this section, some definitions and basic properties of the Sugeno integral which will be used in the next section are presented.

### Definition 2.1

(Fuzzy measure [7, 19])

Let Σ be a *σ*-algebra of subsets of *X*, and let $\mu: \Sigma\rightarrow[0,\infty[$ be a non-negative extended real-valued set function. We say that *μ* is a fuzzy measure iff:

(FM1): $\mu(\varnothing )=0 $;
(FM2): $E,F\in\sum$ and $E \subseteq F$ imply $\mu(E)\leq\mu (F)$ (monotonicity);
(FM3): $E_{n} \in\Sigma$ ($n \in\mathbb{N}$), $E_{1} \subset E_{2} \subset\cdots$ , imply $\lim_{n \rightarrow\infty} \mu(E_{n})= \mu(\bigcup_{n= 1}^{\infty} E_{n})$ (continuity from below);
(FM4): $E_{n} \in\Sigma$ ($n \in\mathbb{N}$), $E_{1} \supset E_{2} \supset\cdots$ , $\mu(E_{1}) < \infty$, imply $\lim_{n \rightarrow\infty} \mu(E_{n})= \mu(\bigcap_{n= 1}^{\infty} E_{n})$ (continuity from above).

Let $(X,\Sigma,\mu)$ be a fuzzy measure space and *f* be a non-negative real-valued function on *X*. We denote by $\mathcal{F}_{+}$ the set of all non-negative measurable functions and by $L_{\alpha}f$ the set $\{x\in X \mid f(x)\geq\alpha\}$, the *α*-level of *f* for $\alpha\geq0$.

### Definition 2.2

Let $(X,\Sigma,\mu)$ be a fuzzy measure space. If $f \in\mathcal {F}_{+}$ and $A\in\Sigma$, then

(i) The Shilkret integral [20] of *f* on *A* with respect to the fuzzy measure *μ* is given by
  $$(Sh) \int_{A} f \,d\mu:=\sup_{\alpha\geq0} \bigl\{ \alpha \cdot\mu (A \cap L_{\alpha}f ) \bigr\} ; $$
(ii) The Sugeno integral [6] of *f* on *A* with respect to the fuzzy measure *μ* is defined by
2 where ∨ and ∧ denote the operations sup and inf on $[0,\infty[$, respectively.

The following theorem gives most elementary properties of the Sugeno integral and can be found in [19, 21].

### Theorem 2.3

*Let* $(X, \Sigma, \mu)$ *be a fuzzy measure space with* $A, B \in\Sigma$ *and* $f, g \in\mathcal{F}_{+}$. *Then*

;
*for a non*-*negative constant* *k*;
3. *If* $f \leq g$ *on* *A*, *then* ;
4. *If* $A \subset B$, *then* ;
;
6. *If* $\mu(A) < \infty$, *then* .

### Remark 2.4

Let $F(\alpha)=\mu(A \cap L_{\alpha}f)$, from parts (5) and (6) of the above theorem, it is very important to note that
 Thus, from a numerical point of view, the Sugeno integral can be calculated by solving the equation $F(\alpha)=\alpha$.

### Remark 2.5

Let $Y\subset\mathbb{R}$ be an arbitrary nonempty interval (bounded or unbounded). Throughout this paper, $Y=[0,1]$ or $Y=[0,\infty[$. Also, we denote the range of *μ* by $\mu(\Sigma)$.

### Definition 2.6

(Generalized Sugeno integral [2])

For a *μ*-measurable function $f:X\rightarrow Y$, we define the generalized Sugeno integral of *f* on a set $A\in\Sigma$ with respect to *μ* and an operator $\Delta:Y\times\mu(\Sigma)\rightarrow Y $ by

3 $$ \int_{A} f \Delta\mu: = \sup_{ y \in Y } \bigl\{ y \Delta\mu (A \cap L_{y} f ) \bigr\} . $$

Let *I* be a real interval and $f : I\rightarrow\mathbb{R} $ be a function. Then *f* is said to be convex (on *I*) provided

$$x,y \in I,\lambda\in[0,1]\quad\Rightarrow\quad f \bigl((1- \lambda)x+ \lambda y \bigr) \leq(1- \lambda) f(x)+ \lambda f(y). $$

Also,

$$S_{f},{}_{x_{0}} (x) = \frac{ f(x) - f (x _{0})}{x - x _{0}} ,\quad x \in I \setminus\{x _{0}\} $$

is said to be the slope of *f* at $x_{0}$.

### Theorem 2.7

(Muresan [22])

*Suppose that* $f : I\rightarrow\mathbb{R} $ *is a convex function*. *Then* $S_{f},{} _{x_{0}} (x)$ *is increasing on* $I\setminus\{x _{0}\} $.

### Theorem 2.8

(Muresan [22])

*Suppose that* $f : I\rightarrow\mathbb{R}$ *is differentiable on* *I*. *Then* *f* *is convex if and only if*

$$f (x ) \geq f (x _{0}) + f ^{\prime}(x _{0}) (x-x_{0}) $$

*for any* $x, x _{0} \in I $.

### Theorem 2.9

(Mitrinović [23])

*Suppose that* $f : [0 , +\infty[\rightarrow\mathbb{R} $ *is a convex function*. *If* *f* *is a non*-*decreasing and continuous function on* $[0 , +\infty[$ *with* $f(0)=0$ *and* $\lim_{x\rightarrow+\infty} f(x)= +\infty$, *then* $f^{-1}$ *exists and has the same characteristics as* *f*.

We say that the operator $\circ: Y \times Y \rightarrow Y $ is non-decreasing if $a \circ c \geq b \circ d $ for $a \geq b $ and $c \geq d$.

### Definition 2.10

(Triangular norm [24, 25])

A triangular norm is a function $T: [0, 1] \times[0, 1] \longrightarrow[0, 1]$ satisfying the following conditions:

($\mathrm{T}_{1}$)
$T(x, 1)= T(1, x)= x$ for any $x \in[0, 1]$;
($\mathrm{T}_{2}$)
*T* is increasing;
($\mathrm{T}_{3}$)
$T(x, y)= T(y, x)$ for any $x, y \in[0, 1]$;
($\mathrm{T}_{4}$)
$T (T(x, y), z )= T (x, T(y ,z) )$ for any $x, y, z \in[0, 1]$.

### Example 2.11

The following operators are *t*-norm:

1. $M (x, y)= \min\{x, y\}$.
2. $\sqcap(x, y)= x \cdot y$.
3. $O_{L} (x, y)= \max\{x+ y- 1, 0\}$.

## Results and discussion

For the classical measure *μ*, the classical Jensen inequality is the following strong property of convex functions (see [26]):

4 $$ H \biggl(\int f(x) \,\mathrm{d}\mu \biggr) \leqslant \int H(f) \, \mathrm{d}\mu, $$

where *f* is *μ*-measurable and $H: [0, \infty[ \longrightarrow[0, \infty[$ is a convex function. The following inequality is known as the discrete Jensen inequality:

$$\begin{aligned} H \Biggl(\sum_{i=1}^{n} \lambda_{i} x_{i} \Biggr) \leqslant\sum _{i=1}^{n}\lambda_{i} H(x_{i}), \end{aligned}$$

where $H: [a, b] \longrightarrow[0, \infty[$ is a convex function, $x_{i} \in[a, b]$ and $\lambda_{i}>0$ for all $i \in\{1, \ldots, n\}$ and $\sum_{i=1}^{n}\lambda_{i}= 1$.

The aim of this section is to characterize the Jensen inequality for the generalized Sugeno integral when *f* is a convex function. Throughout this section, let $(X, \Sigma, \mu)$ be a fuzzy measure space.

### Theorem 3.1

*Assume that* $H : Y\rightarrow Y $ *is a differentiable convex function and* $\circ,\star: Y \times\mu(\Sigma)\rightarrow Y $ *are non*-*decreasing operators satisfying the following conditions*:

1. $a \star0 = a \circ0 = 0$;
2. $H (0 ) = 0$ *and* $H ^{\prime}(y)\geq1 $ *for all* $y \in Y$;
3. $\int_{A} f\star\mu\in Y $ *for an arbitrary set* $A \in\Sigma $ *and a measurable function* $f:X \rightarrow Y$.

*Then the Jensen inequality*

5 $$ \int_{A} H (f)\circ\mu\geq H \biggl(\int_{A} f\star\mu \biggr) $$

*is sharp if and only if*, *for any* $y \in Y $ *and* $b \in\mu(\Sigma)$,

6 $$ H(y)\circ b \geq H (y \star b). $$

### Proof

*Sufficiency*. Let $y\in Y$. Since *H* is a differentiable convex function, by Theorem 2.8 we have

$$H (t )- H (y )\geq H ^{\prime}(y) (t - y ) $$

and by assumption (2),

$$H (t )- H (y )\geq(t - y ) $$

for all $t \in Y$. So we have

$$H \bigl(f(x) \bigr)- H (y )\geq f(x) - y $$

for all $x \in X$. Therefore, by the monotonicity of *μ*, we deduce

7 $$ \mu \bigl(A \cap L_{H(y)}H(f) \bigr) \geq\mu (A \cap L_{y} f ) $$

for an arbitrary set $A \in\Sigma$. On the other hand, since $H(Y)\subseteq Y$, we have

8 $$\begin{aligned} \begin{aligned}[b] \int_{A} H(f)\circ\mu&= \sup_{ a \in Y} \bigl\{ a \circ\mu \bigl(A \cap L_{a} H(f) \bigr) \bigr\} \\ & \geq\sup_{ a \in H(Y)} \bigl\{ a \circ\mu \bigl(A \cap L_{a}H(f) \bigr) \bigr\} \\ &= \sup_{ y\in Y} \bigl\{ H(y)\circ\mu \bigl(A \cap L_{H(y)}H(f) \bigr) \bigr\} . \end{aligned} \end{aligned}$$

Combining (7) with (8) and using the monotonicity of ∘, we get

9 $$\begin{aligned} \begin{aligned}[b] \int_{A} H(f)\circ\mu&\geq\sup_{ y\in Y} \bigl\{ H(y)\circ\mu \bigl(A \cap L_{H(y)}H(f) \bigr) \bigr\} \\ & \geq\sup_{ y\in Y} \bigl\{ H(y)\circ\mu (A \cap L_{y}f ) \bigr\} \\ &\geq\sup_{ y\in Y} \bigl\{ H \bigl(y\star\mu(A \cap L_{y}f) \bigr) \bigr\} . \end{aligned} \end{aligned}$$

Now, we define $h(y ):= y\star\mu (A \cap L_{y}f ) $ for each $y \in Y$. From (9) we have

10 $$ \int_{A} H(f)\circ\mu\geq\sup_{y \in Y} H \bigl(h(y) \bigr). $$

By the assumption, $h (y )\in Y $ and $\sup_{ y \in Y } h (y ) : = \int_{A} f\star\mu\in Y$.

Since *H* is continuous and $H (0 ) = 0$, then the function $S_{H} ,{} _{0}(t)$ (i.e., the function $S_{H} ,{} _{0}: H \setminus\{ 0\} \rightarrow\mathbb{R}$; $t \rightarrow\frac{ H (t)}{t}$) is increasing. From $h(y _{ n })\uparrow\sup_{ y\in Y} h(y )$ we get

$$\begin{aligned} \lim_{n \to+\infty} H \bigl(h(y _{ n }) \bigr) &\leq\lim _{n \to +\infty} S_{H} ,{} _{0} \bigl(h(y _{ n }) \bigr) h(y _{ n }) \\ &\leq\lim_{n \to+\infty} S_{H} ,{} _{0} \bigl(h(y ) \bigr) h(y ) \\ &= \lim_{n \to+\infty} H \bigl(h(y ) \bigr) = H \bigl(h(y ) \bigr). \end{aligned} $$

So,

$$H \Bigl(\sup_{ y\in Y} h(y) \Bigr) = H \Bigl(\lim _{n \to+\infty} h(y _{ n }) \Bigr)= \lim_{n \to+\infty} H \bigl(h(y _{ n }) \bigr)\leq\sup_{ y\in Y} H \bigl(h(y) \bigr) $$

for all $y \in Y$. Consequently,

$$\lim_{n \to+\infty} H \bigl(h (y _{ n }) \bigr) \leq\sup _{ y\in Y} H \bigl(h(y) \bigr). $$

So, we conclude that

$$\begin{aligned} \int_{A} H(f)\circ\mu \geq& \sup_{ y\in Y} \bigl\{ H \bigl(y\star \mu(A \cap L_{y} f) \bigr) \bigr\} = \sup _{ y\in Y} H \bigl(h(y) \bigr) \\ \geq& H \Bigl(\sup_{ y\in Y} h (y) \Bigr)= H \biggl(\int_{A} f\star\mu \biggr). \end{aligned}$$

*Necessity*. Inequality (5) is satisfied for any arbitrary set $A \in\Sigma$ and any measurable function $f:X \rightarrow Y$; in particular, for $f(x )= y \chi_{A} (x ) $ with $y \in Y$, inequality (5) is true. At first, we define $f (x ):= y \chi_{A} (x ) $ with $y \in Y $ and $A \in\Sigma$. By Theorem 2.2 in [2], we have

11 $$ \int_{A} f \star\mu = \max \Bigl\{ \sup_{ a \leq y} \bigl\{ a \star \mu(A) \bigr\} ,\sup_{ a > y} \bigl\{ a \star\mu(\varnothing) \bigr\} \Bigr\} = y \star\mu(A). $$

On the other hand, the assumption $a \leq H(y)$ shows that if $x \in A$, then $H(f(x)) = H(y)\geq a$. So

$$A \subseteq \bigl\{ x | H \bigl(f(x) \bigr) \geq a \bigr\} , $$

and hence $A \cap L_{a} H(f)= A$. Also, the assumption $a > H(y)$ shows that if $x \in A $, then $H(f(x)) =H(y)< a$ and $y S_{H},{} _{0} (y) =H(y) < a $, and hence $S_{H},{} _{0} (y) < \frac{a}{y}$. Thus,

$$A \cap \bigl\{ x | H \bigl(f(x) \bigr)\geq a \bigr\} = \varnothing , $$

because otherwise there exists $x \in A $ such that $H (f(x) )\geq a$. So $y S_{H},{} _{0} (y)= H(y)\geq a$, and hence $S_{H},{} _{0} (y) \geq\frac{a}{y}$, which is a contradiction (note that the slope function is non-decreasing). Now, by the monotonicity of $y \longmapsto y \circ b$ and the conditions $a \circ0 = 0 $ and $H (0 )= 0$, we have

12 $$\begin{aligned} \begin{aligned}[b] \int_{A} H (f ) \circ\mu& = \sup_{ a \in H(Y)} \bigl\{ a \circ\mu \bigl(A \cap L_{a} H(f) \bigr) \bigr\} \\ &= \max \Bigl\{ \sup_{ a \leq H(y) } \bigl\{ a \circ\mu \bigl(A \cap L_{a} H(f) \bigr) \bigr\} , \\ &\quad \sup_{ a > H(y) } \bigl\{ a \circ\mu \bigl(A \cap L_{a} H(f) \bigr) \bigr\} \Bigr\} \\ &= \max \Bigl\{ \sup_{ a \leq H(y)} \bigl\{ a \circ\mu(A) \bigr\} , \sup _{ a > H(y)} \bigl\{ a \circ\mu (\varnothing) \bigr\} \Bigr\} \\ &= \max \bigl\{ H(y) \circ\mu(A ) , 0 \bigr\} \\ &= H(y) \circ\mu(A) \end{aligned} \end{aligned}$$

as $H(y) \circ\mu(A) \in Y \subseteq[0 , \infty]$. Therefore, combining (5), (11) and (12), we conclude that

$$H(y)\circ b \geq H (y \star b). $$

 □

We now investigate for which functions $H : Y \rightarrow Y $ the assertion of Theorem 3.1 is valid if either $Y=[0,1]$ or $Y=[0,\infty[$, $\mu(\Sigma)= Y$ and the operators ⋆,∘ are chosen from the following *t*-norms:

$$\begin{aligned} &a \vee b := \min(a , b),\qquad a.b : = a b\quad \mbox{and} \quad a\oplus b = (a+b-1)_{+}\\ & \quad \mbox{for }a , b \in[0,\infty [,\mbox{ where }(x)_{+}= \max(x , 0) . \end{aligned} $$

### Corollary 3.2

*Suppose that* $Y \subseteq[0 , \infty[ $ *and* $H : Y\rightarrow Y $ *is a differentiable convex function such that* $H (0 )= 0$ *and* $H ^{\prime}(y)\geq1 $ *for all* $y \in Y$. *For an arbitrary subset* $A \in\Sigma$ *and a measurable function* $f : X \rightarrow Y $ *such that* $\int_{A} f\star\mu\in Y$, *the Jensen inequality for the Sugeno integral*
13
*is fulfilled iff*

$$\begin{aligned} H (y \wedge b) \leq H(y)\wedge b, \quad y , b \in Y. \end{aligned}$$

### Proof

Let ⋆,∘ = :∧. Clearly
 and
 Based on Theorem 3.1, the proof is obvious. □

### Corollary 3.3

*Suppose that* $Y=[0,1]$ *and* $H : Y\rightarrow Y $ *is a differentiable convex function such that* $H (0 )= 0$ *and* $H ^{\prime}(y)\geq1 $ *for all* $y \in Y$. *For any measurable subset* $A \subseteq Y $ *and any measurable function* $f : X \rightarrow Y$, *the Jensen inequality for the Shilkret integral*

$$(Sh) \int_{A} H (f ) \,d\mu\geq H \biggl((Sh) \int_{A} f\,d\mu \biggr) $$

*is fulfilled iff*

$$\begin{aligned} H (y \cdot b) \leq H(y)\cdot b, \quad y , b \in Y. \end{aligned}$$

### Proof

Choosing the operators ⋆,∘ = :⋅, we get

$$\begin{aligned} \int_{A} H (f ) \circ\mu= \int_{A} H (f ) \cdot\mu = \sup_{ a \in H(Y)} \bigl\{ a \cdot\mu\bigl(A \cap L_{a} H(f)\bigr) \bigr\} = (Sh) \int_{A} H (f ) \,d\mu \end{aligned}$$

and

$$\begin{aligned} \int_{A} f \star\mu= \int_{A} f \cdot\mu = \sup_{ a \in Y } \bigl\{ a \cdot\mu(A \cap L_{a} f) \bigr\} =(Sh) \int_{A} f \,d\mu. \end{aligned}$$

Based on Theorem 3.1, the proof is obvious. □

### Corollary 3.4

*Suppose that* $Y=[0,1]$ *and* $H : Y\rightarrow Y $ *is a differentiable convex function with* $H (0 )= 0$. *For any measurable subset* $A \subseteq Y $ *and any measurable function* $f : X \rightarrow Y$, *the Jensen inequality*

$$\int_{A} H (f) \oplus\mu\geq H \biggl(\int_{A} f \oplus\mu \biggr) $$

*is fulfilled iff*

14 $$ H \bigl((y + b - 1)_{+} \bigr) \leq \bigl(H(y)+ b -1 \bigr)_{+}, \quad y , b \in Y. $$

### Proof

Let $Y=[0,1]$ and ⋆,∘: = ⊕. Then (6) takes the form (14). Since *H* is differentiable, we have

$$H (y + b - 1) \leq H(y)+ b -1, $$

and so

$$\frac{H(y)-H (y + b - 1)}{1 - b} \geq1 $$

for $0 < b $, $y < 1$. Hence, taking the limit as *b* approaches 1, we have $H ^{\prime}(y)\geq1 $. Now, by Theorem 3.1, we obtain the assertion of this corollary. □

In the next theorems, the sufficient and necessary conditions for the reverse of inequality (5) are given. The proofs are similar to the proof of Theorem 3.1 and are omitted.

### Theorem 3.5

*Let* $\circ,\star:H(Y )\times\mu(\Sigma)\rightarrow\mathbb{R}$ *be arbitrary operators and* $H : Y \rightarrow\mathbb{R}$ *be a differentiable concave function such that* $Y \subseteq H(Y)$, $H (0 ) = 0$ *and* $H ^{\prime}(y)\leq1 $ *for all* $y \in Y $ *and satisfies the following condition*:

- *For all* $y \in Y $ *and* $b \in\mu(\Sigma)$, $H(y)\circ b \leq H (y \star b )$.

*If* $\int_{A} f\star\mu\in Y $ *for* $A \in\Sigma$ *and a measurable function* $f : X \rightarrow Y$, *then*

$$\int_{A} H (f)\circ\mu\leq H \biggl(\int_{A} f\star\mu \biggr). $$

### Theorem 3.6

*Let* $H : Y\rightarrow Y $ *be a differentiable concave function such that* $H (Y )= Y$, $H (0 ) = 0$ *and* $H ^{\prime}(y)\leq1 $ *for all* $y \in Y$. *Let* $a \star0 = a \circ0 = 0$ *for any* $a \in Y$, *and the functions* $y \longmapsto y \circ b$ *and* $y \longmapsto y \star b$ *are non*-*decreasing*. *For an arbitrary set* $A \in\Sigma$ *and a measurable function* $f : X \rightarrow Y $ *such that* $\int_{A} f \star\mu\in Y$, *the Jensen inequality* $\int_{A} H (f)\circ\mu\leq H(\int_{A} f\star\mu) $ *is sharp iff* $H(y)\circ b \leq H (y \star b )$ *for any* $y \in Y $ *and* $b \in\mu(\Sigma)$.

## Generalized Sugeno integral and discrete Jensen inequality

In this section, we deal with the discrete Jensen inequality for the generalized Sugeno integral of convex functions.

### Theorem 4.1

*Let* $(X, \Sigma, \mu)$ *be a fuzzy measure space*. *Let* $f \in\mathcal {F}_{+}$ *and* $A= \{x_{1}, \ldots, x_{n}\}$ *be a finite subset of* *X* *with* $f(x_{1}) \geqslant f(x_{2}) \geqslant\cdots\geqslant f(x_{n})$. *If* $H : Y\rightarrow Y $ *is a differentiable convex function with* $H (0 ) = 0$ *and* $H ^{\prime}(y)\geq1 $ *for all* $y \in Y$, $\lambda_{i}= \mu (\{x_{1}, \ldots, x_{i}\} )$ *for all* $i \in\{1, \ldots, n\}$ *and* $\circ,\star: Y \times\mu(\Sigma)\rightarrow Y $ *are non*-*decreasing operators satisfying the following conditions*:

1. $a \star0 = a \circ0 = 0$,
2. $\int_{A} f\star\mu\in Y$,

*then*

$$\begin{aligned} H \biggl(\bigvee_{1 \leqslant i \leqslant n} \bigl(f(x_{i}) \star \lambda_{i} \bigr) \biggr) \leqslant\bigvee _{1 \leqslant i \leqslant n} \bigl(H \bigl(f(x_{i}) \bigr) \circ \lambda_{i} \bigr). \end{aligned}$$

### Proof

According to Theorem 3.1,

15 $$ H \biggl(\int_{A} f\star\mu \biggr) \leq \int_{A} H(f) \circ\mu. $$

For the left-hand side of (15), by the hypotheses of the theorem, we have

16 $$\begin{aligned} \begin{aligned}[b] H \biggl(\int_{A} f\star\mu \biggr)&= H \biggl(\bigvee _{y \in Y} \bigl(y \star\mu (A \cap L_{y} f ) \bigr) \biggr) \\ & = H \biggl(\bigvee_{1 \leqslant i \leqslant n} \bigl(f(x_{i}) \star \mu \bigl(\bigl\{ x \in A: f(x) \geq f(x_{i})\bigr\} \bigr) \bigr) \biggr) \\ & = H \biggl(\bigvee_{1 \leqslant i \leqslant n} \bigl(f(x_{i}) \star \lambda_{i} \bigr) \biggr). \end{aligned} \end{aligned}$$

For the right-hand side of (15), by using the order $H(Y) \subseteq Y$, we get

17 $$\begin{aligned} \begin{aligned}[b] \int_{A} H(f) \circ\mu&= \bigvee_{a \in H(Y)} \bigl(a \circ\mu \bigl(A \cap L_{a} H(f) \bigr) \bigr) \\ &= \bigvee_{H(y) \in H(Y)} \bigl(H(y) \circ\mu \bigl(A \cap L_{H(y)} H(f) \bigr) \bigr) \\ & \leq\bigvee_{1 \leqslant i \leqslant n} \bigl(H \bigl(f(x_{i}) \bigr) \circ\mu \bigl(\bigl\{ x \in A: H \bigl(f(x) \bigr) \geq H \bigl(f(x_{i}) \bigr) \bigr\} \bigr) \bigr) \\ &= \bigvee_{1 \leqslant i \leqslant n} \bigl(H \bigl(f(x_{i}) \bigr) \circ\lambda_{i} \bigr). \end{aligned} \end{aligned}$$

From (15), (16) and (17) we conclude that

$$\begin{aligned} H \biggl(\bigvee_{1 \leqslant i \leqslant n} \bigl(f(x_{i}) \star \lambda_{i} \bigr) \biggr) \leqslant\bigvee _{1 \leqslant i \leqslant n} \bigl(H \bigl(f(x_{i}) \bigr) \circ \lambda_{i} \bigr). \end{aligned}$$

 □

## Conclusion

The classical Jensen inequality is one of the most important results for convex (concave) functions defined on an interval with a natural geometrical interpretation. In order to obtain a characterization for the classical Jensen inequality for the generalized Sugeno integral, it is clear that the classical conditions must be changed. Previously, some equivalent conditions have been obtained by losing the basic condition of convexity and replacing this condition with monotonicity (see [2]). This paper studied the Jensen inequality for the generalized Sugeno integral by maintaining the condition of convexity. Also, the necessary and sufficient conditions for the validity of the discrete form of the Jensen inequality for the generalized Sugeno integral were investigated. For further investigations of integral inequalities in the area of the generalized Sugeno integral and their applications in other sciences, the results of this paper will be useful and effective.
